# Mid-level providers in emergency obstetric and newborn health care: factors affecting their performance and retention within the Malawian health system

**DOI:** 10.1186/1478-4491-7-14

**Published:** 2009-02-19

**Authors:** Susan Bradley, Eilish McAuliffe

**Affiliations:** 1 Centre for Global Health, Trinity College Dublin, 3-4 Foster Place, Dublin 2, Ireland

## Abstract

**Background:**

Malawi has a chronic shortage of human resources for health. This has a significant impact on maternal health, with mortality rates amongst the highest in the world. Mid-level cadres of health workers provide the bulk of emergency obstetric and neonatal care. In this context these cadres are defined as those who undertake roles and tasks that are more usually the province of internationally recognised cadres, such as doctors and nurses. While there have been several studies addressing retention factors for doctors and registered nurses, data and studies addressing the perceptions of these mid-level cadres on the factors that influence their performance and retention within health care systems are scarce.

**Methods:**

This exploratory qualitative study took place in four rural mission hospitals in Malawi. The study population was mid-level providers of emergency obstetric and neonatal care. Focus group discussions took place with nursing and medical cadres. Semi-structured interviews with key human resources, training and administrative personnel were used to provide context and background. Data were analysed using a framework analysis.

**Results:**

Participants confirmed the difficulties of their working conditions and the clear commitment they have to serving the rural Malawian population. Although insufficient financial remuneration had a negative impact on retention and performance, the main factors identified were limited opportunities for career development and further education (particularly for clinical officers) and inadequate or non-existent human resources management systems. The lack of performance-related rewards and recognition were perceived to be particularly demotivating.

**Conclusion:**

Mid-level cadres are being used to stem Africa's brain drain. It is in the interests of both the government and mission organizations to protect their investment in these workers. For optimal performance and quality of care they need to be supported and properly motivated. A structured system of continuing professional development and functioning human resources management would show commitment to these cadres and support them as professionals. Action needs to be taken to prevent staff members from leaving the health sector for less stressful, more financially rewarding alternatives.

## Background

Access to emergency obstetric care is a key indicator of health system performance as well as the fundamental right of any woman. Over the past decade maternal mortality in Malawi increased dramatically and has reached 1,800 maternal deaths per 100,000 live births [1]. The proportion of women attended by a professional during delivery has stagnated at around 57% and the lifetime risk of maternal death, estimated at 1:7, is one of the highest worldwide [2]. Strengthening human resources (HR) capacity and improving the environment for safe motherhood are key priorities [2-4].

In countries with the worst human resources crises, additional, country-specific cadres have been developed and deployed for many years to address priority needs, such as access to emergency obstetric care. These mid-level providers (MLPs) are defined as cadres of health care workers who undertake roles and tasks that are more usually the province of internationally recognised cadres, such as doctors and nurses, but whose pre-service training is usually shorter and who possess lower qualifications [5]. In Malawi they include clinical officer, medical assistant, registered nurse/midwife, nurse midwife technician and enrolled nurse/midwife grades.

Clinical officers carry out the bulk of major emergency obstetric operations, with figures as high as 93% in government hospitals and 78% in mission facilities, and have postoperative outcomes comparable to those of doctors [6,7]. Given that caesarean sections are the commonest surgical procedure performed in Africa [7] significant maternal and neonatal deaths are being averted by the work of these mid-level providers. Yet Christian Health Association of Malawi (CHAM) establishment and staffing figures for 2007 showed a 77% vacancy rate for this crucial cadre (personal communication, 26^th ^June 2007). More health care workers, equitably distributed and with sufficient skills of the right mix to address context-specific health needs, are urgently needed [8,9].

Health worker performance is a crucial element of a successfully functioning health system and has an evident impact on quality of care. Boosting performance is seen as a crucial step in encouraging recruitment and retention in low-income countries [10]. Performance relies on internal motivation but the presence of external factors, such as the necessary skills, intellectual capacity and physical resources to do the job, clearly have an impact [11]. While there have been several studies addressing retention factors for physicians and registered nurses [12,13] data and studies addressing the perceptions of these mid-level cadres on the factors that influence their performance and retention within health care systems are scarce [5]. There are major practical implications for exploring mid-level cadres' perceptions of these factors.

This study set out to explore the perceptions of mid-level providers regarding the factors affecting their performance and retention within the Malawian health system and addressed the following questions:

• What factors affect the quality of their working environment?

• What structures and systems exist for MLP support and supervision?

• What possibilities do MLPs feel are available for training and career progression?

• What incentives and motivation do they feel are currently in place or are needed for them to remain within the health care system?

• How do MLPs feel that more established health care personnel view them?

## Methods

Qualitative health research aims to answer "how" and "why" questions [14]. It is already clear that MLPs are leaving health systems or failing to be recruited in the first place. However, information is lacking on the specific factors that can encourage retention and improve performance for this particular subset of health care providers in this specific context. The best way to understand these issues is to directly explore the perceptions and views of the personnel involved.

An exploratory qualitative study was designed to provide an opportunity for MLPs to examine their experiences and identify the issues that confront them collectively as well as individually. Focus group discussions were carried out using homogeneous groups to allow the development of an analysis based on commonality of experience and to reduce problems of organizational hierarchy or status factors inhibiting discussions. Medical grade groups included clinical officers (CO) and medical assistants (MA). Nursing grade groups included registered nurses/midwives (RN/M), nurse midwife technicians (NMT) and enrolled nurses/midwives (EN/M). A discussion guide was generated using factors emerging from the literature to shape the content. An exploration of a range of data collection tools from previous studies helped to shape the format. The guide addressed the key research questions, yet was flexible enough for participants to suggest their own priorities and solutions.

Key informant interviews with strategic personnel were used to develop an understanding of extra dimensions of the research questions that became apparent during data collection. These semi-structured interviews allowed in-depth exploration of specific issues, as well as providing additional background and context.

### Sample and setting

The study took place in four rural mission hospitals in Malawi in July 2007. The study population of interest was 'MLPs of emergency obstetric and neonatal care currently employed in mission facilities'. A purposive sample was planned, with all staff members from these cadres within selected hospitals invited to participate. Staff members who had volunteered to participate and who were free on the day of the visit were included in the study.

All interviews took place in English, as Malawi is an Anglophone country in which education takes place in English and any members of the mid-level cadres have undergone at least 12 years of formal education [5]. Participants were fully apprised of the purpose of the research, assured of confidentiality and asked to provide written informed consent.

Four nursing grade focus groups took place (n = 18, 3 × male, 15 × female). Three medical grade focus groups and one in-depth interview took place (n = 12, 7 × male, 5 × female). Interviews were conducted with six key informants with expertise in human resources, training or administration, who were identified in an iterative process during the study.

### Data analysis

A thematic framework was used to analyse the data. This was developed through a deductive process of top-down coding based on *a priori *themes identified in the literature review [5,15-18] and inductive, bottom-up coding based on key themes emerging from the raw data. These codes were systematically applied to the data set in an indexing exercise, allowing the data to be summarised by theme. The next step in the data analysis was a charting exercise. The original research questions and emerging themes from the data provided headings that were used to rearrange data and to summarise it according to thematic content. This process allowed the researcher to start to identify the range and relationships between concepts, leading to a mapping and interpretation exercise that identified the key dimensions of the research questions [19,20].

## Results

Seven thematic areas were identified in the framework analysis. These were job descriptions; management and supervision; training and career progression; incentives and retention factors; resource constraints; motivation; and status with other health care providers. Cross-cutting these thematic areas was the perception that MLPs are not being supported in their work (Table 1).

**Table 1:** Perceptions that MLPs are not being supported

***Thematic area***	***Voices of key informants and focus group discussion participants***
Job descriptions	"We as nurses, we know what to do as we have learnt from school. But, eh, the job description given by the hospital, we are not given." (FGD, ENM1)
	"...it's up to the CHAM institutions to adopt or adapt them...to fit them according to their working environment." (KI, HRO1)
	"You can't say no, because as I was saying there is a critical shortage of staff" (FGD, CO1)

Management and supervision	"...it's not like something which is constant or regularly done. It's erratic. I think it is a problem. It matters. Because if you could have something constant you know for sure that at one point I will have this. But then you are not sure, so all the time you are working it's like, 'I don't know what will happen next,' so you are in constant fear sort of or you are not stable." (FGD, RNM1)
	"Even if the anaesthetists can say, 'we don't have this drug for anaesthesia.' You complain to the Administrator, they will tell you we don't have money. But it's an essential thing, which a medical personnel would know, that this is essential." (FGD, CO6)
	"If one is given appraisal so one is being encouraged for her contribution. So most of the time no, there's no appraisal." (FGD, CO6)
	"...appraisal would be a very important issue, because it would be motivating you to work even more." (FGD, NMT3)
	"But here, somebody does well, no difference. Does bad, no difference." (FGD, CO5)

Training and career progression	"...if you join that facility as a nurse you work the rest of your life at that grade." (KI, HRO1)
	"Look at me, working at the same position for 11 years." (FGD, NMT1)
	"So a policy that a clinical officer remains a clinical officer, there's no motivation, there's no what." (FGD, CO7)
	"I don't want to die a clinical officer. I am still young. I would like to increase my knowledge, my skills and to be a somebody." (FGD, CO4)
	"...as a medical assistant at least I have hope of becoming a clinical officer in the future. But what happens to me if I become a clinical officer?" (FGD, MA1)
	"Money is not a good motivator, it's not the perfect motivator. People still need to have the job satisfaction in terms of their career path." (KI, Admin1)
	"...then people would get motivated. They would feel like they are real professionals in their field, rather than just having a diploma..." (KI, Admin2)

Incentives and retention factors	"The problem you see, when it comes to remuneration package, it's discriminatory, let's be honest. Doctors are favoured...100% of the time it is COs, MAs, who are running the hospitals in Malawi...It's very unfair." (KI, HRO1)
	"If you've worked for it you need to have something to show...the pay, the package, is too little for the work that we do." (FGD, CO6)
	"The allowances we are given for taking calls, it's horrible, it's almost a joke. And it's a flat figure, it's fixed, but we do more hours, sometimes get so exhausted. In the past at some point they tried to make the allowance according to the hours that you put in. But then they have seen that the figures which were coming out..." (FGD, CO1)
	"Here the biggest problem is of salaries, because comparing our salaries to untrained persons, to untrained personnel, there's not much difference." (FGD, ENM3)
	"But here though I think accommodation is good, but we don't have enough houses. That's why they are failing to employ more staff, because of shortage of houses." (FGD, RNM3)
	"Once they have employed you, it's over." (FGD, NMT5)

Resource constraints	"You still want to manage each and every day that patients are seen, but you are few of you, therefore you strain yourself." (FGD, CO4)
	"Our problem is lack of...many inadequate equipment and materials to use... due to maybe the financial stand of institutions...they will still insist that you still use those things. You are always improvising." (FGD, NMT3)
	"The next is you lose a patient because you cannot access the blood." (FGD, CO5)
	"The nurses are shortage...Providing there is somebody attending the patients, but the quality of the nursing itself is not...it's not good as we were trained." (FGD, NMT5)
	"A lot of things are against us. It's a difficult situation working here." (FGD, CO1)
	"All these problems which I meet I should not encounter." (FGD, NMT4)

Motivation	"We sacrifice ourselves to be working, even during all the hours, even beyond our levels, only to make sure that we want to assist those who want to be assisted." (FGD, CO3)
	"...we get afraid of being sacked. So we are trying to do our work best." (FGD, NMT2)

Status with other health care providers	"...with the advent of College of Medicine and the coming in of our own doctors that we train, it's like the medical assistant and the clinical officer, they have sort of been like relegated to the background." (FGD, CO1)
	"Students (registered nurses) are sometimes rude at you, because they see you like someone with low grade. And they think after graduating they will be above you, so when you are instructing them at times they are rude to you, because they already know that you are under them. They can't respect you...in spite of you having that big experience." (FGD, NMT1)
	"I suspect if you look for the signals that we have, I think you can think maybe they don't appreciate our services the way they treat us some of the times... They (medical officers) feel like they can do without us." (FGD, CO8)

Individual voices identified by grade and number

FGD = focus group discussion, KI = key informant interview

### Job descriptions

Only 17% of those interviewed had written job descriptions. These did not always resemble actual duties undertaken and often had discretionary components, such as "...any job assigned to you." This lack of clarity, combined with chronic staff shortages and staff working across different wards and departments, means some staff members exceed their scope of practice (SOP). This was much less likely for nursing grades but far more frequent in facilities with the worst staff shortages. Staff members use their discretion to decide whether they have the necessary skills, with CO1 saying they must "...look inside yourself to find out if you are able to do this, if it is within your capabilities."

### Management and supervision

There was broad-based concern about the role, skills and transparency of management, with the overwhelming impression of ad hoc, erratic implementation. Managers were perceived to not know what staff members are doing, nor to have the necessary skills to work in a multidisciplinary arena.

"It is like a very similar individual, he is taking care of...say maybe medical side. He is also taking care of management side, he is also taking care of financial side. Of which everything is under that very same person. When in fact maybe this one is not, eh, competent in all those fields." (NMT3)

Most staff members reported receiving support from senior colleagues when necessary, but supervision was reported to be extremely limited and almost exclusively negative or corrective in nature. A Matron suggested that this is due to staff shortages, as high nurse-patient ratios lead to mistakes, while there is lack of officer-grade staff with adequate training to carry out the supervisory role. This has a major impact on staff appraisal, with the majority of staff members reporting the absence of any such mechanisms in their institution.

### Training and career progression

Both nursing and medical cadres feel they have been trained up to a level where they are useful, then left there. Staff voiced concerns about management bias in the selection of staff for promotion, the lack of performance-based promotion and the fact that when promotions are made they happen erratically or are constrained by quotas.

Career progression and expanding SOP are relatively straightforward for nursing cadres. Providing their school qualifications are adequate, a route exists to take them from certificate level to degree and beyond. However, progression moves staff members away from patients and into managerial roles, or makes them unaffordable or outside the designated skill mix for the hospital. "And then when you have a Masters that sees you out of the ward for good." (RNM2)

Career progression for COs is a major problem. Training should fill gaps in the system and in HR terms "...for COs the immediate gap they should fill is the doctor..." (Training Officer). However, there is no direct route from CO to doctor. COs now follow a four-year programme to qualify for a diploma in clinical medicine, yet to become a doctor a CO must start the Medical Bachelor and Bachelor of Surgery programme (MBBS) in the College of Medicine "...from zero, like somebody coming from high school." (CO5) The only alternatives are a degree in health education or biomedical science, or a Master of Public Health degree. All these options are described as wasting COs' skills and experience, while the education or public health options move them out of clinical practice. A Training Officer interviewed agrees the current route for COs "...does not link them straight to their career; it doesn't."

One Administrator's opinion is that anyone should be able to progress and have a clear career path "...but with clinicians, once they get their diploma they are stuck. I think the government should look critically at the career path for clinical officers. It's a major problem." One attempt to address this issue is the sponsoring of a two-year CO surgical skills course by CHAM. This is seen as part of a process that would provide entry to a career path that could lead to a degree and ultimately the MBBS. However, there is currently no certification or monetary reward for completion of this course and any proposed route for COs is still uncertain. COs themselves are keen on the idea of certificate courses, specifically for clinical officers, which would allow them to specialise in disciplines such as paediatrics or surgery, but would prefer these to be at degree level.

### Incentives and retention factors

Staff members were asked to identify the three most important factors that need to be changed, introduced or improved to ensure they would remain in the health sector. It is clear that medical cadres have very specific requirements: better financial incentives, improved opportunities for career development/education and improved management/communication. Other incentives did not rank very high. Nursing cohorts, however, have much more variable requirements. While career progression and salary were main concerns they did not score as high as for medical grades. Factors such as improved physical and human resources, improved management/communication, accommodation, provision of free uniforms and hot meals for night staff were important too.

Both groups strongly agreed that improvements in access to and funding for education, upgrading and promotion would be a major incentive. This echoes exit interviews conducted by CHAM which suggest that offering continued education would retain staff. Staff members also want to be encouraged with performance-related rewards, based on regular assessments, for those doing better, working longer hours or taking on added responsibility. The current lack of recognition is demotivating. "...even if you put in extra effort it's fixed, so you decide not to put in any effort at all." (CO6) Management agree that hospitals need to introduce a system in which remuneration is tied up with performance.

There was robust consensus that access to the Internet would be a big incentive, particularly for medical grades. Currently Internet access is reserved mainly for office staff and there is clearly an impression that management does not view MLP use of the Internet as work.

"...we are in a changing world whereby medicine is dynamic. You need to be updated. You need to have the latest information on HIV, latest information on doing caesarean sections, and all this would be seen on the Internet. To compare and contrast what other people are doing and what you are doing so that you can improve." (CO7)

### Resource constraints

Human and material resource constraints have the biggest impact on the working environment. Staff shortages lead to exaggerated working hours, heavy workloads, lack of "off duties" and more frequent night shifts. Lack of material resources adds to workloads by causing time-consuming struggles to improvise and can negatively affect patient outcomes or increase length of stay. The shortage of protective clothing and gloves is a worry to all cadres, particularly in the context of HIV.

"...sometimes we are called to see a patient who is bleeding. And let's say we have to remove a placenta, and this is what we call manual removal. Now we don't even have gloves that are long enough, because we are supposed to have gloves that are up to our elbow because you have to put your whole hand in. There's nothing. So you think, 'can I leave this patient to bleed?' No. You still have to carry on to do the procedure." (CO6)

### Motivation

One of the drivers for staff to work outside their SOP and to remain in post despite difficult circumstances is the desire to help their fellow Malawians. "One of the reasons why we work so hard...is because we know people are suffering in the villages. And if they come they want our assistance" (CO2); and "The main goal is to serve the community, people's lives." (CO3)

Most staff cited a good team dynamic, where staff members provide each other with feedback, support and cover, as having the most positive impact on their ability to do their job. Other positive elements are good patient outcomes, caring for the sick, patient gratitude and working with the local community. Medical grades also valued working in a challenging environment and gaining experience.

### Status with other health care providers

Tensions between COs and doctors came across very strongly in the medical grade interviews. COs expressed frustration and anger at the "huge" differential in salary, benefits, workloads and status between COs and doctors. This cadre feels invisible and unappreciated. There was resentment that COs work hard but doctors "...will leave you behind, toiling" (CO1) while they upgrade and specialise. Only CO4 expressed an alternative opinion, saying she did not feel any tensions. *"*In reality, yes, a doctor is more than a clinical officer."

One senior CO described COs as a "crucial cadre" who, unlike doctors, will work in rural settings and who essentially run the health system. The lack of recognition of their qualifications, both at home and internationally, was contrasted with registered nurses, who are internationally mobile.

"If we are to make progress at all the gap between the doctors and the clinical officers should be narrowed down, both in what they do as well as in the career grade. The career path should be more smooth, because there is a break somewhere. Because you are a clinical officer and the next lad is what? Is a doctor." (Administrator)

Nursing grades do not seem to suffer as much from hierarchy and status factors as medical cadres.

## Discussion

One of the most significant findings from this study is the predicament of COs, who are in the invidious position of being a cadre without a career path. They are described as crucial to the running of the health system, yet there is a widespread perception that they have been trained to a level at which they are useful, and then abandoned. Although there are some possibilities for in-service training such extra effort is not recognised or rewarded, and in some instances attracts increased, but unpaid, responsibilities.

The current system constrains the development of COs to the extent that they either stagnate, leave clinical practice or opt out of the health sector entirely. This has the potential for a significant negative impact on quality of patient care. COs themselves reported their lack of motivation. Staff members who are trapped are unlikely to provide the best service [15].

Frustrations with this situation are not limited to COs. Administrators bear the brunt of trying to recruit from an already scarce cadre of staff, then have to deal with the disaffection among COs who feel stuck in a system that appears not to value them or want them to progress. The predicament of COs reflects a larger issue that cuts across all cadres studied: the waste of human capital. Throughout this study highly trained, experienced staff expressed their frustration at knowing they could develop and "be more", but of not having any encouragement or opportunity to do so. Since attracting staff to rural facilities is a major problem, it makes sense to support those who are already in post.

The impact of inadequate human resources management (HRM) at the facility level is another key component of the findings of this study and confirms previous work done [16-18]. Integrated HRM is one of the key elements in addressing the HR crisis [21]. Participants in this study did not have job descriptions, frequently exceeded their scope of practice, received minimal or negative supervision and did not know how well they were performing.

In three of the four hospitals visited, a supportive team environment seemed to substitute for formalised HRM. This may well be a consequence of the Christian ethos and family environment fostered in mission facilities.

There is a clear call from staff for supportive supervision and increased transparency, accountability and consistency in the application of HRM. At the moment there is little incentive for staff to work harder or perform at a higher level, because any extra effort or skill is not recognised, nor can it be measured. Indeed, the lack of recognition or reward has a negative effect, demotivating staff and leading to suboptimal performance.

It is also clear that financial incentives are necessary but not sufficient for motivation. Salary and allowance differentials among different cadres of staff and across facilities appear to have a particularly demotivating effect. The variability between mission institutions leads to poaching between facilities, while tensions are caused between cadres when some qualify for an allowance, such as a responsibility allowance, that another group feels they too deserve. Eventually allowances come to be seen as an entitlement and no longer serve as an incentive [22]. Because of the critical shortage of doctors COs find themselves expected to assume this responsibility and role, but without the corresponding benefits. For these reasons a performance-based system of enhancing staff salaries may prove more effective in motivating and improving performance.

## Conclusion

COs are a crucial element of emergency obstetric care in Malawi, providing the bulk of clinical care at hospital level. It is clear that women's access to life-saving interventions would be severely constrained without them, yet they find themselves trapped in terms of career progression, unsupported in their work and unappreciated for the contribution they make. Further research is needed to provide an evidence base for their role and impact on health outcomes and to determine the appropriate skill mix necessary to render equitable, high-quality care [23].

Improvement in HRM is crucial to provide clear, consistent messages about what is expected from MLPs and what they can expect in return. There should be concerted efforts to create a positive upward spiral, where staff are supported and supervised and improved performance is recognised and rewarded. Using continuing professional development as a non-financial incentive can increase motivation, but also feeds into the loop by improving skills and performance. There is a clear connection between a functioning HRM system, performance-related rewards and continuing professional development.

It is a dangerous strategy to be complacent about the role of MLPs as an answer to the brain drain of health professionals. Those who suggest that these cadres have a role in tackling the human resources crisis also caution that quality of care will suffer if they are not properly supported and motivated [5,9,24]. A structured system of continuing professional development, management and supervision would show commitment to these cadres, support them as professionals and send a clear message about their value and worth to the health system.

### Limitations

Data for this study were collected from only a small number of mission hospitals, so may not be representative of the entire Malawian health care system and there may be regional variations. Findings from this study, however, have been compared with other research in the field and an attempt made to examine the relationships between this and other MLP populations with a view to generalizability.

The unavailability of health care staff, either due to work schedules or absolute numbers, meant it was necessary to undertake small focus groups or to mix cadres of health providers or genders. This was not an ideal situation as small groups, gender dynamics or hierarchy issues may inhibit group interaction.

The study took place in a different culture, using the English language. Although all participants had been extensively educated in English it was clear that for some of them this was a difficult second language. Nor can one make assumptions about shared understanding or usage of language [14]. Undoubtedly the researcher missed cultural differences in non-verbal behaviour.

## Competing interests

The authors declare that they have no competing interests.

## Authors' contributions

SB and EM participated in the design and analysis of the study. SB conducted the research and drafted the paper. All authors contributed to the final manuscript. All authors read and approved the final manuscript.
